# Epigenetic signatures of genetic risk in Alzheimer’s disease

**DOI:** 10.1002/alz.090389

**Published:** 2025-01-03

**Authors:** Sarah Marzi

**Affiliations:** ^1^ UK Dementia Research Institute, London United Kingdom

## Abstract

**Background:**

Microglia are key players in Alzheimer’s disease (AD): Genetic risk for AD is enriched in microglial enhancers, and microglial gene regulatory networks have been shown to be disrupted in AD. Here, we studied polygenic and variant‐specific (APOE) risk burden for AD in a xenotransplantation model of AD and human post‐mortem brain tissue.

**Method:**

We profiled gene regulation by RNA‐seq and ATAC‐seq in human iPS‐derived microglia, xenotransplanted into the APPNL‐G‐F mouse model of AD. We used isogenic APOE2/3/4 and APOE knockout (KO) microglia. In parallel, we purified microglial nuclei from a large cohort of human post‐mortem brain samples with and without AD neuropathology. We performed nanobody H3K27ac CUT&Tag on these nuclei to characterize the AD‐associated microglial enhancer landscape.

**Result:**

We observe numerous gene regulatory differences across APOE microglia in the chimeric mice. From E2 to E3 and E4 we see increased expression and regulation of pro‐inflammatory cytokine signalling. Conversely, the potentially protective disease associated and antigen‐presenting response microglia states are downregulated. Human post‐mortem microglia show widespread AD‐associated acetylation differences. By leveraging genetic variants in the CUT&Tag sequencing data, we were able to directly link genetic variants and epigenetic signatures, identifying potential mechanisms underlying non‐coding genetic risk. Finally, we show parallels between APOE‐linked epigenetic responses in human post‐mortem samples and the xenotransplantation model.

**Conclusion:**

We identified gene regulatory mechanisms underlying microglial activation in neuropathological environments in chimeric mice and human post‐mortem brain samples. Our findings are important for understanding the upstream epigenetic mechanisms governing microglial activation in neurodegenerative disease, as well as directly linking these to genetic risk factors of disease.

